# Iodine-Doped Graphene Oxide: Fast Single-Stage Synthesis and Application as Electrocatalyst

**DOI:** 10.3390/ma15176174

**Published:** 2022-09-05

**Authors:** Adriana Marinoiu, Daniela Ion-Ebrasu, Amalia Soare, Mircea Raceanu

**Affiliations:** National Institute for Cryogenics and Isotopic Technologies ICSI-Rm, Valcea, ICSI Energy, Uzinei Street, No. 4, 240050 Ramnicu Valcea, Romania

**Keywords:** graphene, iodine-doped graphene, fuel cells

## Abstract

Iodine-doped graphene oxide is attracting great attention as fuel cell (FC) electrocatalysts with a high activity for the oxygen reduction reaction (ORR). However, most of the reported preparation techniques for iodine-doped graphene (I/rGO) could be transposed into practice as multiple step procedures, a significant disadvantage for scale-up applications. Herein, we describe an effective, eco-friendly, and fast technique for synthesis by a microwave-tuned one-stage technique. Structural and morphological characterizations evidenced the obtaining of nanocomposite sheets, with iodine bonded in the graphene matrix. The ORR performance of I/rGO was electrochemically investigated and the enhancement of the cathodic peak was noted. Based on the noteworthy electrochemical properties for ORR activity, the prepared I/rGO can be considered an encouraging alternative for a more economical electrode for fuel cell fabrication and commercialization. In this perspective, the iodine-based catalysts synthesis can be considered a step forward for the metal-free electrocatalysts development for the oxygen reduction reaction in fuel cells.

## 1. Introduction

Iodine-doped graphene oxide is considered to have an increased technological capability as a catalyst for the reduction reaction of oxygen (ORR) in fuel cells. Nowadays, fuel cells (FCs) are thought to be one of the most highly performant electrochemical-based systems that are using fuel (hydrogen) electrochemical reduction to obtain electrical energy, for both mobile and stationary applications. The increased importance of fuel cell utilization for energy production applied in a large number of systems confirms the idea that electricity produced by fuel cells could be considered a rapidly emerging progress in the age of energy, ready for commercialization. One such solution is the proton exchange membrane fuel cell (PEMFC) technology, which definitely has the capability of becoming a sustainable and clean electricity source. PEMFC shows the most promise due to the demonstrated high efficiency and low pollution [1,2]. Low-temperature PEMFC exploitation is currently the latest technology, particularly for light vehicles or stationary applications. The use of the noble metal Pt as the favoured catalyst for both electrodes is one of the impediments that limits the PEMFC commercialization as the Pt catalyst is the highest contributor to the overall cost of a fuel cell. Due to the significantly lower kinetics occurring at the cathode for the oxygen reduction reaction (ORR), important research has been dedicated to developing advanced ORR catalysts. Implementing a high-performance and durable platinum-free catalyst, especially for ORR, could significantly enable the large-scale commercialization of fuel cell-powered vehicles [3,4,5,6]. The technological achievements, understandings, and membrane assembly electrodes (MEA) integration activities of ORR catalysts have received important attention, and up-to-date literature tries to cover all aspects regarding the catalyst activity, durability and MEA performance.

On the other hand, since graphene’s discovery as another allotrope form of carbon, it has been proven that it has remarkable physical–chemical properties for many electrochemical systems, such as fuel cells, electrolyzers, solar cells, etc. Graphene is known as a single-layered graphite composed of a one-atom-thick carbon sheet constituting a hexagonal lattice structure. Graphene oxide represents the oxidized form of graphene. Graphene oxide (GO) has attracted interest as a suitable route to produce graphene. The presence of different functional groups leads to a hydrophilic behaviour that is strongly influenced by the oxidation level. The GO sheets indicate a good dispersion capacity resulting from a strong electrostatic charge and hydrophilicity. The reduction of GO is a common route to obtain a graphene-like structure. Different chemical, thermal, or photo-thermal reduction methods can be used to prepare rGO structures. Depending on the methods used, the rGO produced can be more or less close to the pure structure of graphene. Reducing agents are either inorganic (sodium borohydride) or organic (phenyl hydrazine hydrate or hydroxylamine) chemical agents. Typically, thermal reduction occurs in an inert atmosphere at high temperatures 300–2000 °C. The photochemical reduction of GO involves a direct laser beam at wavelengths below 390 nm [7]. The promising properties of GO and rGO derivatives, such as the high specific surface area, the electrical conductivity of rGO, and the chemical activity of GO, are mainly useful for energy storage applications.

In order to make graphene active from an electrochemical point of view, it is necessary to change the carbon structure by its functionalization with different types of heteroatoms such as halogens. Currently, the halogen-doped graphene presents high catalytic activity for the ORR reaction [8,9,10,11,12,13]. Additionally, by functionalization it is possible to create defects within the graphene structure with the final scope being the improvement of their electrochemical performances towards the oxygen evolution reaction, as well as fuel cells mechanical and thermal stability both in an acidic and alkaline environment.

Between halogens, the iodine forms diatomic molecules, where two iodine atoms share a pair of electrons to obtain a stable octet; at high temperatures, these diatomic molecules reversibly dissociate a pair of iodine atoms. Similarly, the iodinated anion, I-, is the strongest reducing agent among the stable halogens, being the lightest oxidized back to the diatomic I2. Iodine is the halogen with the largest atomic radius in the group, it can form partially ionized bonds that can promote charge transfer. The ORR activity can be improved through electrocatalytic efficiency due to iodine doping, facilitated by the formation of load transfer complexes (I3- and I5-) that increase the doping capacity and functionality of the graphene support.

To dope the graphene-based materials with iodine atoms is a difficult target that brings a high level of difficulty from a synthesis point of view, since the iodine atomic radius is bigger compared with the carbons’ making it more difficult to transfer the charge from one surface to another. However, the negative ion I3- is formed during the iodine graphene doping process. This will enhance the number of positives in the graphene structure and improve ORR’s functionalized graphene catalytic activity. In this context, it should be noted that during iodine doping bonds are formed between iodine and the graphene matrix that can transport the charge between the halogenated iodine groups.

Furthermore, due to the high potential of the halogen-doped graphene to link O_2_, the O–O bonds of the adsorbed oxygen are reduced, hence enabling effective transformation to water after reduction and protonation.

Most of the reported synthesis methods of iodine-doped graphene are multiple step procedures, a significant disadvantage for scale-up applications. Having this target, several works have been developed. Iodine-functionalized graphene nanoplatelets for ORR could be prepared by using sequential steps of ball milling for 24 h. The co-doping of iodine with ammonia on the GO surface was accomplished at a high temperature of over 950 °C. Iodine-doped rGO could be obtained by mixing graphite oxide with PI3 followed by refluxing for 24 h. High-quality I/rGO was reported by using a ball milling time of 48 h and further freeze-drying for 48 h. Iodine-doped graphene was prepared by thermal annealing at temperatures between 500 and 1100 °C. Another method involved a high-efficient heat-pressing technique to obtain I/rGO nanosheets [14]. Our recent work reported an iodination method by electrophilic substitution starting from graphite and various oxidation agents, in the presence of different catalysts such as AlI3 [8]. These reported I/rGO-based materials exhibited good ORR electrochemical properties and energy storage. However, the mentioned materials required high temperatures, a long reaction time as well as unsafe dopants and hazardous reagents. Thus, it is essential to improve the chemical process from an environmental point of view through a facile route to prepare iodine-doped graphene in mild reaction conditions (shorter time and lower temperature). In this respect, this paper aims to develop an unprecedented simple and low-time consuming method to synthesize the iodine-doped graphene oxide. Microwaves act directly via dielectric loss more than heat convection encountered in the conventional heating process; thus, a rapid heating as well as selective heating is expected. This is regarded to be promising in shortening the reaction time and yielding hot spots with a high temperature. To the best of the authors’ knowledge, this paper is the first attempt to employ the microwave method to produce such materials. One of the advantages of the microwave heating procedure utilization is the time and energy processing reduction. Briefly, during the cost-effective microwave process, the energy is directly supplied to the material surface through an electromagnetic field. This leads to a fast drying of the material with decreased thermal gradients. The MW field and the dielectric response of the material provide the ability to increase the temperature by using microwave energy.

The microwave (MW) process has been widely applied in many chemical syntheses; however, this research is the first of its type where it is applied for the iodine doping process together with the reduction of graphene oxide, by using a simpler, more effective, faster, and more economical protocol at atmospheric pressure. Through this paper, a canvas-like structure I/rGO was synthesized starting from the graphene oxide (GO) by involving the MW route under mild conditions. This work emphasizes the capability of I/rGO to work as an ORR electrocatalyst used for fuel cells electrodes development, as a low-cost alternative to the commercial platinum-based catalysts.

## 2. Experimental

### 2.1. Catalyst Synthesis

The volatiles from the commercial graphene oxide (GO) powder (Abalonix, Norway) were removed by degassing at 110 °C. GO was then was added into demineralized water (DW) and stirred for 15 min. The hydroiodic acid (HI) (Sigma-Aldrich, St. Louis, MO, USA, 55 wt.%) was completed and the solution was mixed by 10 min sonication. This compound was further reduced with sodium borohydride (NaBH_4_, Scharlau, 98%), and the reaction mixture was ultrasonicated (80 Hz) for 10 min. The obtained solution was spilled in the microwave reactor (MARS 6 One touch, CEM) cylindrical vessels. The single-stage microwave (MW) iodine-doping process parameters, in terms of temperature and reaction time, were optimized for mild conditions: atmospheric pressure, temperature 40–60 °C, power 800 W, and reaction time 15 min. The product was chilled moderately to room temperature. The obtained black solid was separated by ultracentrifugation, and then, alternately, washed using DW and ethanol (Chimreactiv, 99.5%). Finally, the product was dried by lyophilization.

The original and economical iodine graphene doping method presented in this paper consisted of: utilization of commercial GO, HI as iodine source, and NaBH_4_ as reducing agent in mild reaction environment in the MW field.

### 2.2. Physical and Chemical Measurements

X-ray photoelectron spectroscopy (XPS) characterization was done using the X-ray photoelectron spectrometer (PHI-5000 VersaProbe, PHI-Ulvac/Physical Electronics). XPS spectra were measured using monochromatic Al Kα radiation (1486.7 eV). The qualitative elemental analysis was undertaken by recording the broad spectra, and the identification of the different types of chemical bonds that are formed on the surface was obtained from the high-resolution spectra deconvolution. The PHI-MultiPak software was used for the XPS spectra interpretation. The quantitative elemental analysis in terms of chemical elements atomic concentration was performed by calculating the peak areas by considering the sensitivity factors of the analyzed elements. The yield of iodine was 95 ± 2% based on XPS analysis data of three separately prepared samples. Microstructural investigation and elemental analysis were performed using a Carl Zeiss FEG SEM equipped with an EDX system. The I/rGO powder was transferred for SEM investigation on a double-sticky carbon tape and the surplus was blown with compressed air. The specific surface area of the I/rGO samples was measured by Brunauer–Emmett–Teller (BET) method, using AutosorbIQ (Quantachrome). BET technique is generally consecrated method to estimate the specific surface area (S_BET_) involving the nitrogen adsorption measurements at 77 K. The adsorption–desorption isotherms were obtained in low-pressure nitrogen atmosphere. Prior to the BET analysis, the I/rGO powder humidity was eliminated under vacuum outgassing at 388 K, for 240 min. The pore size distribution and total pore volume were calculated by Barrett–Joyner–Halenda (BJH) method and was applied to evaluate the porous surface of iodine-doped graphene as well as of starting graphene oxide. The mass changes with temperature of the reduced graphene oxide (rGO) and iodine-doped samples (I/rGO), respectively, were studied by thermogravimetric analysis (TGA) using TGA-DSC e NETZCH STA 449 F5 Jupiter system. The TGA measurements were done in air flow, in the temperature domain of 27–1000 °C, heating the sample with a rate of 2 K min^−1^. In order to prove the iodine doping of the reduced graphene oxide support, ATR-FTIR method was used. The Frontier FT-NIR spectrometer was used for the attenuated total reflectance Fourier-transform infrared spectroscopy (ATR-FTIR) characterization. The infrared spectra were recorded in transmittance, in domain of 650–4000 cm^−1^.

### 2.3. The Electrochemical Measurements

Oxygen reduction reaction (ORR) of the iridium-doped samples (I/rGO) was measured in comparison with non-doped reduced graphene (rGO). The measurements were carried out in commercial glass cell with three electrodes using a 2273 potentiostat (Princeton Applied Research). A controlled speed OrigaTrod rotating disk electrode (RDE) with 0.196 cm^2^ glassy carbon (GC) disk was used as working electrode (WE). A 3 M Ag/AgCl (Metrohm) was used as reference electrode (RE) and a platinum wire as counter electrode (CE). All the measurements were done alkaline electrolyte (0.1 M KOH) that was flushed with oxygen for one hour before each experiment. The pH of our 0.1 M KOH aqueous electrolyte in which KOH salt is dissolved is 13, that means a strong base solution. This electrolyte concentration (pH) is commonly used for the catalysts’ electrochemical properties characterization (ORR, OER HER, and HOR), and it was chosen according to the most published papers in the domain. The potential presented in this work are versus Ag/AgCl RE. The catalyst aliquot was prepared by stirring overnight 5 mg rGO-based powder together with 240 µL isopropyl alcohol, 10 µL DW, and 20 µL 5 wt.% Nafion^®^ solution. For measurements, 7 µL of catalyst solution was deposited onto GC surface, that was previously polished with alumina and diamond paste, cleaned with demineralized water and dried in air.

In order to calculate the number of electrons (*n*) transferred during the ORR process, linear sweep voltammograms were recorded with 5 mV/s, between 0.4 V and −0.7 V and at various rotation speeds (ω) (among 250–1000 rpm). Linear sweep voltammetry (LSV) curves for I/rGO were plotted as *j*, which is the measured current density (mA^−1^ cm^2^_geo_) vs. ω^−1/2^ (rad s^−1^)^−1/2^ and studied following Koutecky–Levich (KL) Equations (1)–(3) [15,16,17].

(1) 1j=1jL+1jk=10.62nFC0(D0)32υ−16ω12+1nFAkC0

(2) $$B=0.62nFC_{0}{\left(C_{0}\right)}^{\frac{3}{2\upsilon }-\frac{1}{6}}$$

(3) $$j_{k}=nFAkC_{0}$$

where: *j_L_*—maximum (limiting) current density required to achieve ORR reaction (A cm^−2^); *j_k_*—kinetics current density (A cm^−2)^; *F*—Faraday constant (96.485 kCmol^−1^); *A*—area of the RDE electrode (0.196 cm^2^); *C*_0_—concentration of oxygen (1.2 × 10^−6^ mol cm^−3^) in 0.1 M KOH electrolyte; *D*_0_—diffusion coefficient of oxygen in the electrolyte (1.9 × 10^−5^ cm^2^ s^−1^); *ν*—kinematic viscosity (0.01 cm^2^ s^−1^) in 0.1 M KOH; and *k*—the rate constant (cm s^−1^) for the electron transfer [16,17].

## 3. Results and Discussion

The main difference between GO and rGO is the number of oxygen molecules present, hence the capability to participate differently into the doping process. Since rGO is obtained from the reduction of GO by thermal, chemical, or electrical treatments, in the rGO there are always some defects and some oxygen functional groups. Basically, different reducing agents could lead to various carbon to oxygen ratios and chemical compositions in rGO.

The research of affordable common routes for the preparation of I/rGO-based materials has seen substantial progress and efforts, especially for the field of ORR electrocatalysis. It is important to state the difference between iodine functionalization and iodine doping to highlight the importance of the method in this work, especially for obtaining graphene-based materials with electrocatalytic properties.

The doping of graphene with iodine offers a proper solution to modify its electronic proprieties. Iodine-doped graphene has been successfully obtained by a simple and cost-effective MW method.

The physical adsorption currently used for graphene-based materials iodine-doping can be considered as a more favorable method, in comparison with the chemical adsorption, due to the enhancement of the charge carriers’ concentrations. During the physical adsorption, the charge carriers’ mobility is not affected, while in the case of chemical adsorption, the chemical dopants covalently bond on the pristine lattice causing crystalline defects that will irreversibly alter the structure.

**Figure 1:**
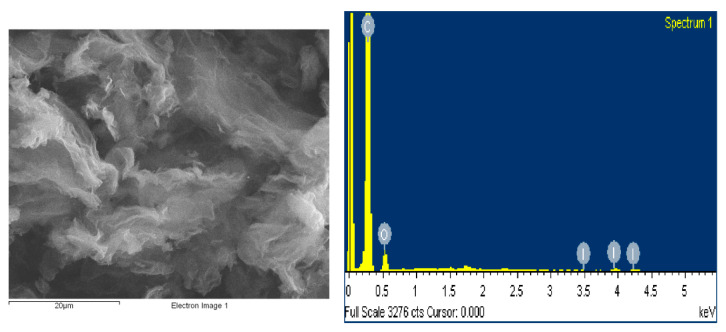
SEM microstructure and energy dispersive X-ray spectroscopy spectrum of prepared I-doped graphene oxide.

The structural and morphological characterization of the iodine-doped graphene was carried out by scanning electron microscopy (SEM). Figure 1 presents the micrograph of the I/rGO that is almost transparent and exhibits micrometer-sized creased bundles formed by the graphene layers overlapping and twisting during the iodine doping process. Thin, crumpled nanosheets are randomly arranged that form a crake-shaped spongy structure and a good connected network that provide an important role in transport properties. In Table 1 are presented the elemental composition, including carbon, oxygen, and iodine. The estimated concentration values of the elements were obtained from the energy dispersive spectroscopy (EDS) measurements. According to this method, the iodine concentration of 1.67 wt.% was obtained.

XPS spectroscopy was used to determine the chemical state of iodine forms corresponding to the I/rGO sample.

**Figure 2:**
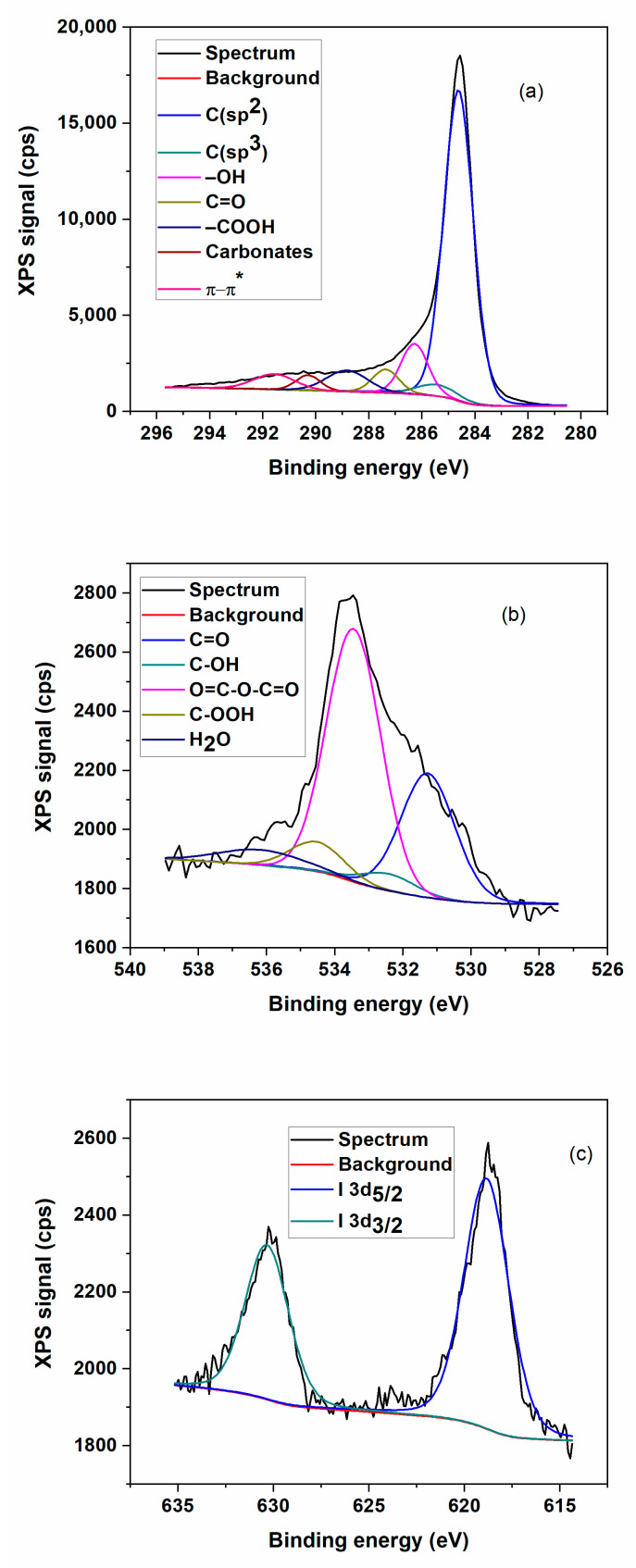
Detailed XPS high resolution survey for I/rGO sample: (**a**) carbon C1s; (**b**) oxygen O1s; and (**c**) iodine I3d.

Figure 2 shows the high-resolution survey scanning of iodine orbitals (I3d_5/2_ and I3d_3/2_) in the 616–635 eV interval. Table 2 summarizes the results obtained from functions fitting after the background subtraction. The presence of over 75% sp^2^ hybridized C–C bonds certifies the efficaciousness of the graphene oxide matrix reduction. The C–C bonds are dominant since the HI acid reduction occurred, because most of the hydroxyl and epoxy groups were removed. However, some oxygen-containing functional groups still remain in the rGO sheets, which are typical for chemically reduced GO. The elemental composition and iodine bonding types were investigated. As presented in Figure 2, the C1s (~284.5 eV) and O1s (~533.7 eV) peaks are clearly observed in the XPS spectra of prepared I/rGO. The corresponding iodine peaks were easily noticed in the high-resolution XPS of I3d (Figure 2c), which can be caused by iodine’s doping concentration of approximately 0.13 atomic%. The presence of I3d peaks at I3d_5/2_ (~618.8 eV) and I3d_3/2_ (~631.3 eV), respectively, suggests the enhancement of the iodine interaction with the graphene oxide matrix. The deconvoluted I3d_5/2_ peak shows two peaks: first is the 617.96 eV peak of triodide (I_3_^−^), and the second is 620.21 eV corresponding to pentaiodide (I_5_^−^). These results are the same with the one reported in literature [7]. The first peak is attributed to the iodine anions from an excess of HI and the second peak is assigned to the introduction of iodine into the GO substrate reduced by HI. These obtained results revealed that iodine atoms were efficiently embodied into the graphene matrix and the I_3_^−^ and I_5_^−^ remained on its surface after the microwave process. According to chemical composition calculated through this method, the iodine concentration of 1.3 wt.% was obtained.

As iodine was used as a dopant, its content was very small, 0.13% in the synthesized doped rGO, and the data were collected with noise, but two Gaussian curves were clearly adjusted to the data with FWHM. The noisy XPS deconvoluted spectrum may be due to the high surface sensitivity of XPS, since it has been reported that iodine is a strong p-type dopant for graphene. The XPS signal-to-noise ratio (C-I) is the height of a signal (peak) above a background relative to the noise in the scan/spectrum measured at some point off the peak.

The mechanism for the adsorption of iodine molecules on the GO has been studied both theoretically and experimentally to adjust the electronic structure of the GO surface. Theoretically, the adsorption of halogen atoms on GO/rGO was investigated by using a semi local function. It was accepted that an iodine atom can accept 0.5 electrons from the carbon substrate. The doping of the rGO by the physical adsorption is interesting since it can increase the concentration of the carriers without affecting carrier mobility as in the case of chemical adsorbed dopants, where the covalent function can produce crystalline defects and irreversibly alter the electron structure [18].

With the view to understand the iodine doping impact towards the structure and porosity of graphene oxide support, further investigations are carried out. One of these is gas adsorption that gives an extensive description of the porous materials in comparison with the specific porosity, surface area, and pore size distribution. From the shape of sorption/desorption isotherms obtained during the BET analysis, it is possible to understand the elementary reactions linked to the sorption and desorption of nitrogen and its comportment in spongy-like solid structures. The BET model is based on the multilayer adsorption of gas on the surface of the adsorbent. The BET method determined the specific surface area through the P/P_0_ domain in the 0.1 and 0.3 (multipoint model). According to this, the linear high-pressure part is suitable for evaluation of the primary mesopore volume and the external surface area. The secondary mesopore volume (which does not form ordered structures) is calculated as a distinction between the total pore volume and the primary mesoporous volume. The model calculates the maximum amount adsorbed at pressures close to the saturation vapour pressure by converting it to the volume of liquid adsorbed, similar to the evaluation of the micropore volume [19].

**Figure 3:**
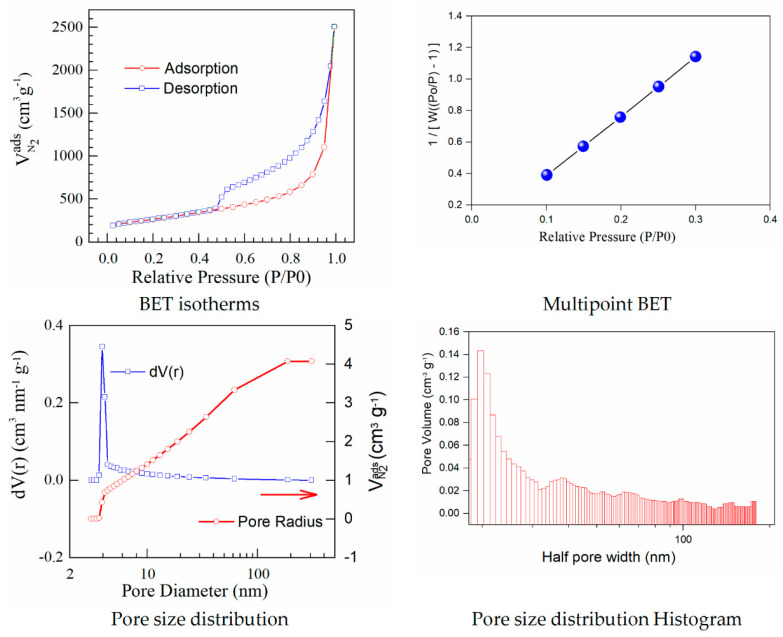
BET isotherms and BJH curves corresponding to graphene oxide.

**Figure 4:**
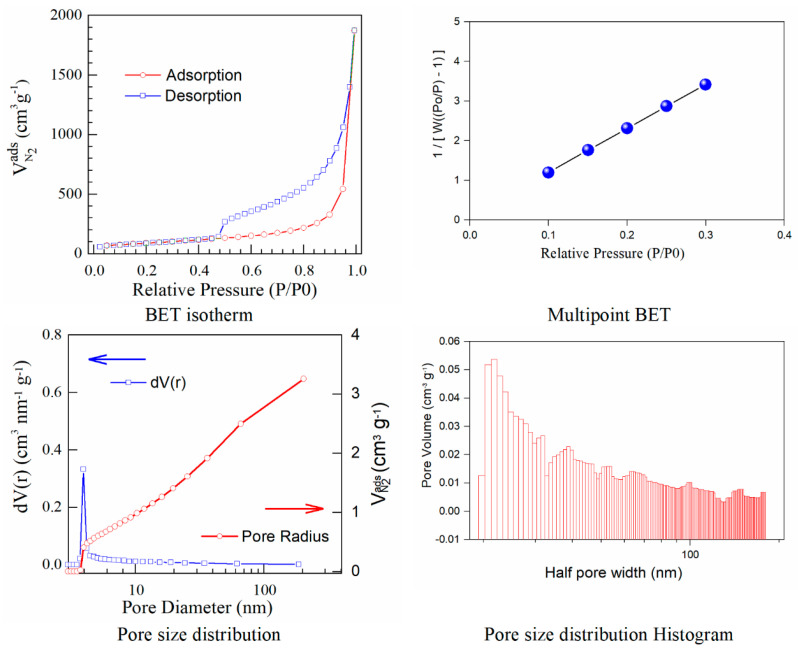
BET isotherms and BJH curves corresponding to iodine−doped graphene.

The results calculated using the isotherms for adsorption/desorption are presented in Figure 3 and Figure 4. The N_2_ adsorption/desorption isotherms for both samples correspond to the type IV isotherm according to the specifications from IUPAC. The micropore volume was calculated in the low-pressure region, as the intercept with the adsorbed amount axis represents the volume adsorbed in micropores. It is important to note the excellent linearity starting from the lowest adsorbed amount and also the correlation coefficient R^2^ very close to one, both indicating a well-defined monolayer and constituting the indicative for the BET data validity.

Both adsorption–desorption isotherms present a typical behavior for a mesoporous structure with a uniform pore size distribution, suggesting that the prepared materials have good transport properties among micropores, mesoporous, and macro-porous channels. The calculated specific surfaces and the estimated textural properties for the pore volumes are presented in Table 3.

Herein, iodine−doped graphene presents a vertical uptake under P/P_0_ = 0.02, and a hysteresis loop from P/P_0_ = 0.45 to P/P_0_ = 1.0, which is due to the co-existence of both micropores and mesopores. Moreover, it can be noticed that there are little ultra-micropores in the doped graphene material, thus the porous material could provide more active sites and thus improve the redox reactions. The pore size is a critical parameter that affects the basic physicochemical properties and applications of porous doped−graphene, but the preparation of I/rGO with a controllable pore size is still a big challenge. In this paper, the model BJH was applied for the estimation of the pore volume distribution (both for rGO and I/rGO) (Vp, (cm^3^ g^−1^)) and the pore size and half pore width (nm). The results clearly indicated that a textural change occurred. According to the BJH, most of the pores from the iodine-doped graphene oxide have a size below 5 nm. The doping of iodine into the graphene network provides the modifications of surface area and also for the pore size of the doped material, according to Table 3. The type H_3_ hysteresis loop is correlated with slit-shaped pores, possibly between parallel layers.

Iodine-doped graphene has a BET surface area (311 m^2^/g) that is significantly more reduced than that corresponding to rGO (922 m^2^/g). We can presume that after the iodine inclusion into the graphene framework, the surface area will decrease as an effect of the doping process that favors the tendency and probability of restacking.

A linear relationship between the pore volume and the relative pressure *P/P*_0_ is presented as a multipoint BET representation. The nitrogen adsorbed volume as a function of *P/P*_0_ was used to calculate the pore size distribution in the form of *dV/dr* as a function of pore radius (*r*). The calculated pore volumes indicated an open stacking order. The iodination process maintains the sheet-like morphology also observed by SEM microscopy. Perhaps the layers become graphitized and this is reflected in the strongly reduced BET surface area. The pore volumes correspond to open 3D staking of reduced graphene oxide-based powders.

**Figure 5:**
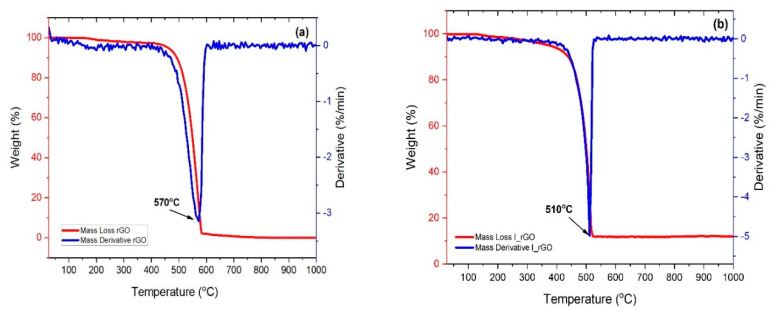
TGA/DTGA plots corresponding to: (**a**) r−GO and (**b**) I/rGO.

The thermal decomposition curves of the reduced graphene and iodine-doped reduced graphene are presented in Figure 5a,b. Both samples present one decomposition step assigned to the reduced graphene degradation. The I/rGO mass loss is not complete (approximately 86%) and starts at approximately 300 °C, in comparison with the rGO complete decomposition that starts at 400 °C. The 16% of undecomposed I/rGO represents the amount of remanent mass of iodine that has a 2446 °C melting point, lower that the temperature range used for the TGA measurements. From the first derivative mass loss (DTG) it can be observed that the I/rGO decomposition temperature is 510 °C, which is 60 °C lower than the undoped reduced graphene oxide, which is due to the partial breaking of C–I bonds.

**Figure 6:**
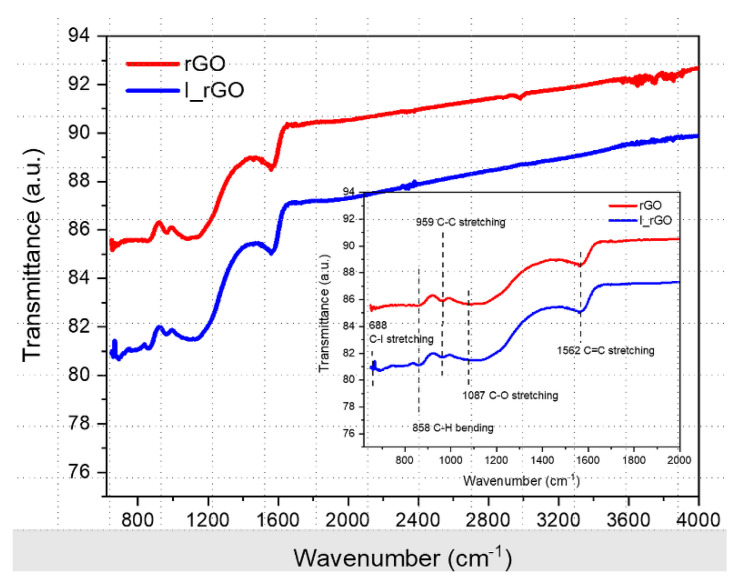
Overlaid ATR−FTIR rGO and I/rGO spectra with the fingerprint zone in the inset.

Figure 6 shows the overlapped ATR-FTIR rGO and I/rGO samples’ spectra, with the fingerprint zone in the inset. Both spectra exhibit the common C=C, C−C, and C−O stretching vibrations and C–H bending vibration assigned to 1562, 1087, 959, and 858 cm^−1^, respectively. Iodine presence in the I/rGO structure can be noticed from the band at C–I assigned to the C−I stretching vibration.

**Figure 7:**
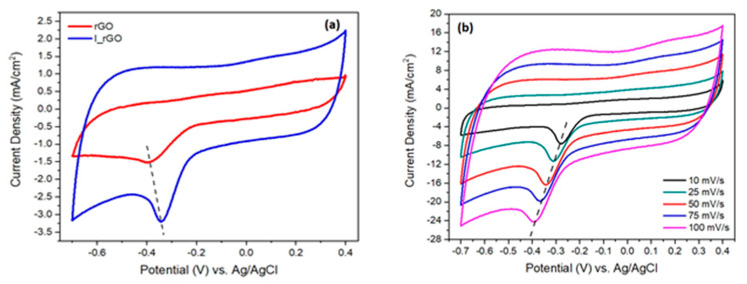
(**a**) Cycling voltammograms of reduced graphene oxide (rGO) and iodine−doped rGO (I/rGO) recorded between 0.4–0.7 V vs. Ag/AgCl, in oxygen atmosphere, scanned with 50 mV/s; (**b**) cycling voltammograms of I/rGO at different sweeping rates.

The catalytic performance of rGO and I/rGO for the oxygen evolution reaction (ORR) was characterized in alkaline electrolyte (0.1 M KOH) using the cycling voltammetry method (CV) at 50 mV/s [12]. In Figure 7 are presented the rGO and I/rGO cycling voltammograms (CVs) scanned with a scan rate of 50 mV/s. Analyzing the I/rGO CV, it can be noticed that the oxygen reduction (ORR) peak from −0.343 V is approximately twice higher (current density of 3.3 mA/cm^2^) in comparison with 1.52 mA/cm^2^ from 0.383 V of rGO, respectively. Moreover, the I/rGO ORR peak position is shifted with 50 mV to a more positive potential. These findings strongly indicate that the iodine-doped reduced graphene oxide have ORR catalytic activity. The influence of the sweeping towards the I/rGO CV shape and ORR current density is presented in Figure 7b. The results show a shift to more oxidative potentials (left side), and an enhancement of the cathodic peak with a sweeping rate, from 7.4 mA/cm^2^ corresponding to 10 mV/s, to 24 mA/cm^2^ associated with 100 mV/s. This comportment is assigned to the I/rGO surface extensive oxidation with the sweeping rate. 

The electrochemical activity can be assigned to the ORR mechanism by taking into account the higher electronegativity of iodine (2.66) than carbon (2.55), polarizing carbon atoms and favoring oxygen adsorption as well as the charge transfer.

**Figure 8:**
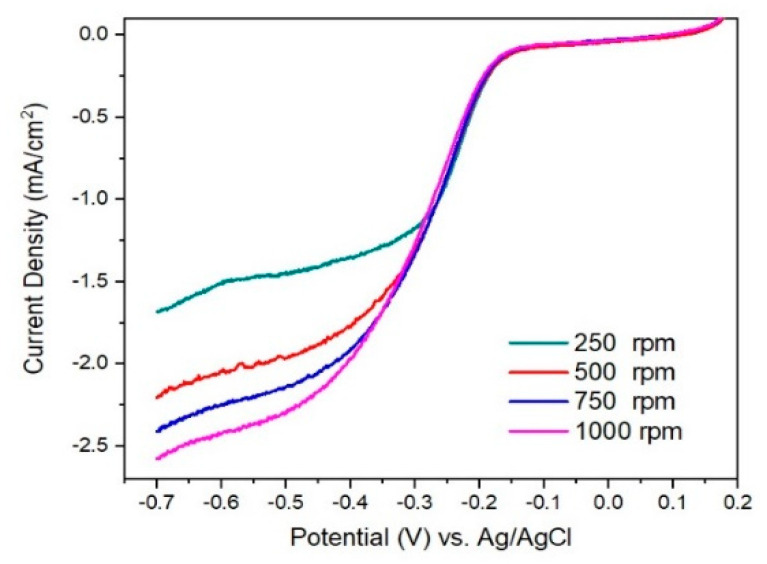
Linear sweep voltammograms for I/rGO at various rotation speeds (250 to 1000 rpm) on RDE.

**Figure 9:**
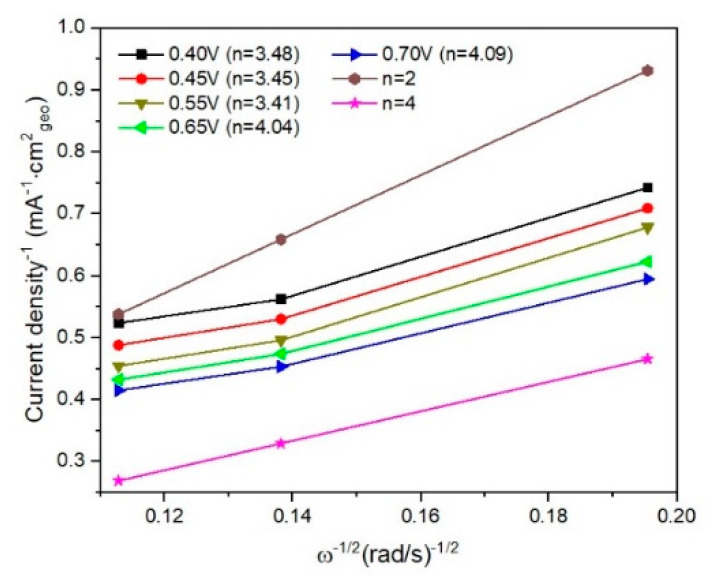
Fitted K–L plots for I/rGO determined from linear sweep voltammograms.

In Figure 8 and Figure 9 are presented linear sweep voltammograms and the Koutecky–Levich (K–L) plots for I/rGO as current density (mA^−1^ cm^2^_geo_) vs. ω^−1/2^ (rad s^−1^)^−1/2^, for various rotation speeds (among 250–1000 rpm) and different potentials (0.4–0.7 V). From the fitted K–L plots, a fair linear relationship at all potentials is noticed, which confirms the electroreduction of iodine. The number of transferred electrons is between 3.41 and 4.09, indicating the preponderance of the four-electron transfer mechanism in the ORR corresponding to the direct reduction of O_2_ to H_2_O [20]. The number of transferred electrons (*n*) was calculated to be in the range of 3.41–4.09, suggesting a predominant four-electron O_2_ reduction in combined two-electron and four-electron pathways of the ORR process [21,22]. At lower overpotentials, the *n* value is around 3.4, indicating that a hydroperoxide anion HO_2_^−^ is formed as an intermediate reduction product of the two-electron reduction of O_2_. The HO_2_^−^ is reduced into OH^−^ at the electrode at more negative potentials, thus restoring the overall four-electron ORR process. The value of *n* slightly over 4 towards −0.70 V vs Ag/AgCl suggests a possible another reduction reaction than ORR occurring in the studied catalytic system [23]. In our case, it is likely that iodine species are involved in such a process [24]. According to the XPS, ATR-FTIR, TGA, and BET, the ORR performance of I/rGO is increased concerning rGO by iodine doping.

## 4. Conclusions

This work indicates that starting from a commercially accessible material—graphene oxide—through a specially designed process for chemical synthesis in the microwave field, iodine-doped graphene with specific morphological and structural properties could be obtained. The single-stage microwave (MW) iodine-doping process parameters in terms of temperature and reaction time, were optimized for mild conditions: atmospheric pressure, a temperature of 40–60 °C, a power of 800 W, a reaction time of 15 min, and a doping concentration of 1.3 wt.% was obtained. Cycling voltammetry and rotation disk electrode polarization methods were employed to study the I/rGO electrocatalytic activity with regard to the ORR. The good ORR activity of the I/rGO probe can be explained by the iodine doping of the rGO matrix, promoted by the formation of load transfer complexes (I^3−^ and I^5−^) that increase the functionalization capacity of the graphene support. Developing a green chemistry method to prepare I/rGO using MW could be a step forward in obtaining affordable electrodes with ORR activity.

## Figures and Tables

**Table 1:** Elemental concentration of the iodine-doped graphene sample.

Element	Weight (%)	Atomic (%)
C K	86.94	90.89
O K	11.39	8.94
I L	1.67	0.17
Total	100.00	100.00

**Table 2:** Chemical composition for prepared iodine-doped graphene.

C1s		O1s		I3d5	
Weight%	Atomic%	Weight%	Atomic%	Weight%	Atomic%
78.84	84	19.86	15.87	1.3	0.13

**Table 3:** Comparative textural properties of rGO and iodine−doped graphene.

Samples	S_BET_ (m^2^ g^−1^)	Pore Volume (cm^3^ g^−1^)	Pore Radius (A)	Mesoporosity %	Macroporosity %
rGO	922	4.072	19.677	95.3	4.7
I/rGO	311	3.256	19.664	88.7	11.3

## Data Availability

Not applicable.
